# Editorial: Toxicity mechanisms, exposure, toxicokinetic and risk assessment aspects of metals, toxic for animals and humans, Volume II

**DOI:** 10.3389/fphar.2022.1107327

**Published:** 2023-01-11

**Authors:** Yanzhu Zhu, Fatma M. El-Demerdash, Xu Yang, Alex Boye, Michel Mansur Machado, Xinwei Li

**Affiliations:** ^1^ Jilin Agriculture Science and Technology College, Jilin, Jilin Province, China; ^2^ Environmental Studies Department, Institute of Graduate Studies and Research, Alexandria University, Alexandria, Egypt; ^3^ College of Veterinary Medicine, Henan Agricultural University, Zhengzhou, Henan Province, China; ^4^ Department of Medical Laboratory Science, School of Allied Health Sciences, College of Health and Allied Sciences, University of Cape Coast, Cape Coast, Ghana; ^5^ Universidade Federal do Pampa, Uruguaiana, Brazil; ^6^ Key Laboratory of Zoonosis, Ministry of Education, College of Veterinary Medicine, Jilin University, Changchun, Jilin Province, China

**Keywords:** toxicity mechanisms, toxicity exposure, toxicokinetic, risk assessment, metals

Pollutants such as copper (Cu), cadmium (Cd), iron oxide nanoparticles (IONPs), and cyclophosphamide (CTX) exert toxicity on all animal species in the ecosystem (Gioia et al., 2011). Cu is an essential trace element in various cellular processes, but excessive levels of Cu are markedly harmful. Cd can exert toxic effects on various microorganisms, plants, and animals in very low quantities. As the first generation of nanomaterials, IONPs can cure iron deficiency in chronic kidney disease. CTX induces immunosuppression; however, the underlying mechanisms of these pollutants need to be further explored.

Quercetin (Que), Panax ginseng C. A. Meyer (PG), Tamarindus indica (TM), coenzyme Q10 (CoQ), strontium (Sr), and selenium-enriched yeast (SeY) have the potential to be used in the treatment of the toxicity. Que is a special subclass of flavonoid and a powerful antioxidant. PG has a variety of ginsenosides that show diverse biological effects on various diseases. As a traditional medicine, TM has anti-inflammatory and analgesic effects. CoQ10 plays a key role in mitochondrial bioenergetics and exerts a natural antioxidant effect. Selenium is a micronutrient that is essential for the proper functioning of all organisms. High doses of Sr induce alterations in mineralization.

Many researchers have focused on this field and have obtained important findings. Habotta et al. reported that SeY reduces the hepatic and renal damage induced by Cu in broiler chickens, and it can be used as a potential feed supplement (Wang et al.). Wang et al. found that ginsenosides alleviate exogenous toxicity and reduce drug toxicities and they could potentially be used as a treatment for toxicity (Han et al.). Han et al. revealed that IONPs accumulated in the macrophage lysosomes and the spleen eliminate the IONPs in the systemic circulation (Attia et al.). Attia et al. found that Cd damages the adipocyte function (Huang et al.). Attia et al. found that Que could alleviate kidney damage and renal-cell apoptosis induced by Cd (Wang et al.). Wang et al. found that QE has an antioxidant effect in BRL-3A cells (Abdelnaby et al.). Abdelnaby et al. found that hepatorenal damage induced by Cd is alleviated by TM or CoQ supplementation (Zheng et al.). Zheng et al. found that immunosuppression in mice induced by CTX could be treated by ginsenoside Rb2 (Liu et al.). Liu et al. found that primary chondrocyte proliferation is promoted by Sr, but primary chondrocyte differentiation is inhibited by Sr (Liu et al.).

This special issue provides new ideas for the application of Que, PG, TM, CoQ, SeY, and Sr in the prevention and treatment of Cu, Cr, IONPs, and CTX toxicity.

